# Accelerometer-based detection of African swine fever infection in wild boar

**DOI:** 10.1098/rspb.2023.1396

**Published:** 2023-08-30

**Authors:** Kevin Morelle, Jose Angel Barasona, Jaime Bosch, Georg Heine, Andreas Daim, Janosch Arnold, Toralf Bauch, Aleksandra Kosowska, Estefanía Cadenas-Fernández, Marta Martinez Aviles, Daniel Zuñiga, Martin Wikelski, Jose Manuel Vizcaino-Sanchez, Kamran Safi

**Affiliations:** ^1^ Department of Migration, Max Planck Institute of Animal Behaviour, Radolfzell, Germany; ^2^ Department of Game Management and Wildlife Biology, Czech University of Life Science, Prague, Czech Republic; ^3^ VISAVET Health Surveillance Center, Department of Animal Health, Complutense University of Madrid, 28040 Madrid, Spain; ^4^ Animal Health Research Centre (CISA, INIA-CSIC), 28130, Valdeolmos, Madrid, Spain; ^5^ Department of Integrative Biology and Biodiversity Research, University of Natural Resources and Life Sciences, Institute of Wildlife Biology and Game Management (BOKU), Vienna, Austria; ^6^ Agricultural Centre Baden-Württemberg, Wildlife Research Unit, Aulendorf, Germany; ^7^ Centre for the Advanced Study of Collective Behaviour, University of Konstanz, Konstanz, Germany

**Keywords:** wildlife disease monitoring, animal sentinel, wild boar, African swine fever, biosignal

## Abstract

Infectious wildlife diseases that circulate at the interface with domestic animals pose significant threats worldwide and require early detection and warning. Although animal tracking technologies are used to discern behavioural changes, they are rarely used to monitor wildlife diseases. Common disease-induced behavioural changes include reduced activity and lethargy (‘sickness behaviour’). Here, we investigated whether accelerometer sensors could detect the onset of African swine fever (ASF), a viral infection that induces high mortality in suids for which no vaccine is currently available. Taking advantage of an experiment designed to test an oral ASF vaccine, we equipped 12 wild boars with an accelerometer tag and quantified how ASF affects their activity pattern and behavioural fingerprint, using overall dynamic body acceleration. Wild boars showed a daily reduction in activity of 10–20% from the healthy to the viremia phase. Using change point statistics and comparing healthy individuals living in semi-free and free-ranging conditions, we show how the onset of disease-induced sickness can be detected and how such early detection could work in natural settings. Timely detection of infection in animals is crucial for disease surveillance and control, and accelerometer technology on sentinel animals provides a viable complementary tool to existing disease management approaches.

## 1. Introduction

Wildlife infectious diseases threaten livestock production, the global market and human health [1]. To reduce the threat of a spillover to humans or livestock, timely detection of diseases circulating in wildlife is required to take rapid action. Current approaches to monitoring infectious wildlife diseases are largely based on surveillance programmes that rely on the collection of animal carcasses to detect pathogens in a particular area [2]. Depending on the trigger to take samples, these surveillance programmes can take a passive (also known as general or scanning) or active (also known as targeted) form (World Organization for Animal Health). The effectiveness of passive and active surveillance depends on (i) the ability to detect sick or dead wild animals and (ii) the ability to identify the pathogens responsible for a particular disease [3]. Although the latter is often technically relatively easy to achieve, finding and detecting sick or dead animals in the wild remains a major challenge because it necessitates a network of motivated field-based stakeholders (e.g. hunters, wildlife rangers) and advanced technologies [4,5]. Furthermore, surveillance programmes lack early detection capacity because a report of a disease outbreak can only be made after carcasses are found and analysed. Therefore, detection and identification processes can be lengthy and slow. Minimizing the time until the first case is detected is critical for some infectious diseases with a rapid clinical course, such as African swine fever (ASF). Early and rapid detection and subsequent reporting of an outbreak are key to the success and efficacy of containment and management measures and for optimizing economic, social and animal welfare costs [6].

An alternative to traditional passive or active surveillance programmes is the sentinel surveillance approach, which relies on real-time monitoring of sentinel animals. Sentinel animals are living organisms that can act as early warning systems by indicating the presence or emergence of specific diseases or environmental hazards in a particular area [7]. Monitoring the health status of sentinel animals can inform us in advance of where and when the next infectious disease outbreak will occur [7–10]. Recent advances and developments in biologging technologies have opened new avenues for implementing sentinel programmes and monitoring the health status of wild animals. Biologgers are animal-attached devices (tags) containing sensors (movement, accelerometers, proximity sensors, magnetometers, gyroscopes, thermometers, salinity sensors, hygrometers, etc.) that allow tracking of animals and sensing of the state and condition of their surrounding environments [11,12]. In the context of early disease outbreaks, accelerometer sensors are of particular interest for uncovering the internal states of animals [13,14] and for detecting sickness behaviour in animals [15–18]. Sickness behaviour marks the transition to a heat- and energy-conserving posture when animals are sick. This behaviour is an evolved adaptation that facilitates the immune response to combat infections [19]. Sickness behaviour is further characterized by a reduction or even loss of appetite and thirst, sleepiness, disorientation, lethargy, depression and seeking an energy-conserving microenvironment. Although accelerometers are widely used in precision livestock farming to detect sickness behaviours and inform herd health and welfare status [16,20–22], sentinel surveillance and accelerometer sensors are rarely used to track and detect wildlife diseases. This is mainly due to (i) a lack of exchanges and interactions between disciplines (e.g. movement ecology, disease ecology, veterinary sciences, and sensor engineering) and (ii) missing inferences between accelerometer-derived metrics and specific animal diseases [7,23].

In this study, we investigated how infectious pathogens alter patterns of animal activity. Wild boar (*Sus scrofa*) was used as the host model for ASF. In Africa, wild suids have co-evolved with the disease and have developed resistance mechanisms [24]. However, outside the African continent, lethality among members of the Suidae family is very high, reaching up to 90% in wild boar populations where the ASFv genotype II is circulating [25]. Consequently, ASF places a tremendous economic burden on developed and developing countries [26,27] and poses a threat to food security and biodiversity in general. At the individual level, the clinical course of ASF in wild boar and domestic pigs is rapid and dramatic with virulent strains. Animals exhibit symptoms such as lethargy, fever, anorexia, and depression within 7–14 days of infection, ultimately resulting in death [28,29]. This rapid clinical course makes wild boar ASF an ideal host disease model for inferring the relative change in animal activity (sickness behaviour) after infection. ASF is on the priority list for animal health diseases in the European Union and the OIE, and there is currently no recognized vaccine.

During an experiment to assess the pathological evolution and transmission of the ASF virus (ASFV) in wild boar, we opportunistically outfitted individuals with accelerometer sensors attached to their ears to measure their activity. Our aims were threefold: (i) to quantify how the disease affects wild boar activity, (ii) to test whether and when we can detect changes in activity associated with disease onset, and (iii) to validate our laboratory-based observations with semi-free and free ranging wild boars. We showed that ASF infection significantly reduced wild boar activity, allowing simple change point statistical methods to be used for the detection of disease onset. Furthermore, we demonstrated that the behavioural signature of an ASF infection appears to be comparable between individuals living under experimental or natural conditions.

## 2. Methods

### (a) Laboratory animals

Between June and July 2021, 14 wild boars three–five months old, obtained from a commercial farm in Andalusia (Spain), were kept in the biosecurity level 3 laboratory (BSL-3) at the Center for Veterinary Health Surveillance (VISAVET, Complutense University of Madrid, Spain). They had ad libitum access to water and food, and laboratory conditions (i.e. 45–60% humidity and temperature between 21 and 23°C) were maintained constant throughout the experiment. The experimental rooms were illuminated from 7.00 to 21.00 local time. The animals were checked daily by veterinarians and technicians who monitored their internal temperature, recorded clinical signs, kept the pen clean, and controlled the feed supply [29]. After two weeks of acclimatization to the experimental boxes, the wild boars were ear-tagged with an accelerometer sensor by authorized members of the VISAVET team. After four weeks, four wild boars randomly selected (hereafter referred to as ‘infected animals') were challenged intramuscularly with 10 HAD∼50 (haemadsorbing dose) of a highly virulent ASFV (Armenia07), and placed in contact with ten animals (hereafter referred to as ‘contact animals’). They were equally distributed in two boxes, one 15 m^2^ and one 11.4 m^2^ large (each box received two infected animals and five contact animals).

**Figure 1.**
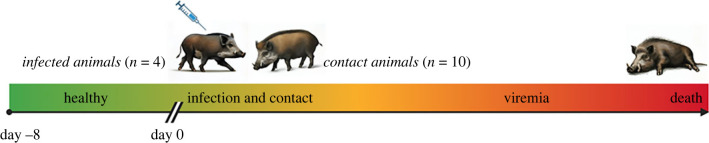
Design used for the original vaccine development experiment showing different phases and timings. Day 0 is the day on which four animals were intramuscularly challenged with 10 HAD∼50 of ASFV Armenia07 and placed in two separate boxes with the contact animals (two infected animals + five contact animals in each box). The healthy phase was considered as the phase between day 0 and day −8; for contact animals, the contact phase was between day 0 and day of first viremia detection, whereas for infected animals, the infection phase was the phase between day 0 and day of first viremia detection. The viremia phase is the phase between the day of the first viremia detection and the day of death for both contact and infected animals.

The day of the first viremia (positive detection of ASF virus in the blood) and the day of death was recorded for all animals. During the viremia phase, the virus can easily access the targeted organs and rapidly affect the host physiology with visible and measurable signs of disease. Based on these days and the infection/contact day, we defined three phases: (i) *healthy* starting 8 days before the experimental infection with ASF virus to day 0; (ii) *infection* from day 0 to the day of the first viremia for infected animals or *contact* from day 0 to the day of first viremia detection for contact animals; and (iii) *viremia* lasting from the day of the first viremia until death (figure 1). Of the 14 individuals, one contact (euthanized after being attacked by the rest of the group) and one infected (eartag lost) were excluded from the analysis.

### (b) Semi-free and free-ranging animals

To compare the activity levels of laboratory animals with those of animals under natural conditions, we deployed similar eartag sensors on semi-free and free-ranging wild boars. Semi-free individuals were captive in a 1 ha large fenced oak-beech forest park in Bad Waldsee, Baden-Württemberg (southern Germany). Ten individuals were tagged at the age of four months and released into the enclosure in June–July 2021. The eartags were recovered by the end of July, once the battery was empty. For sensors deployed in the laboratory, accelerometers recorded a 10-second burst every 4 min at 16 Hz, from which overall dynamic body acceleration (ODBA) was derived similarly (electronic supplementary material, figure S3).

Data for free-ranging individuals were obtained from Donau-Auen National Park, Lower Austria. The National Park is 9600 ha large, containing 65% riparian forest, 15% meadows and 20% water habitats. In autumn 2021, 20 individuals from different groups (fifteen aged 4–12 months, four animals 12–24 months, and one animal 24–36 months) and locations within the park were trapped and marked with accelerometer eartags. Eleven tags were recovered (three recaptured and eight hunted) and included in the analysis (electronic supplementary material, figure S4).

### (c) Accelerometer data

The prototype sensors developed by the Max Planck Institute of Animal Behaviour recorded triaxial acceleration (*X*, *Y* and *Z* axes) in bursts of 10 s every 4 min (range ± 2*g*; sampling frequency 16 Hz). From the raw accelerometer data, we derived the ODBA (in units of gravitational acceleration, *g*). ODBA was calculated by removing the static acceleration (i.e. gravitational acceleration) from the dynamic acceleration (due to animal movement) and summing the dynamic acceleration over all three axes [30]. Static acceleration was calculated using a 2-second running mean applied to the raw acceleration in each axis. ODBA is a valid proxy for animal energy expenditure due to movement [30–32]. As ASF implies high energetic costs for infected animals with marked lethargy [19], we hypothesized that ODBA constitutes a good candidate metric to describe the effect and course of this disease in wild boars.

Owing to battery constraints in the wild, the tags deployed in natural conditions on semi-free and free-ranging wild boar had different accelerometer settings, recording a 4-second burst at 1 Hz every 4 min. Therefore, for these animals, the ODBA was derived at the burst level without applying a running mean. To avoid bias due to animal handling in both captive and natural settings, we only considered the data between one day after tag deployment and one day before tag removal.

### (d) Data analysis

First, we tested ODBA as a relevant metric for detecting the course of ASF infection in wild boars by applying segmented models over ODBA time series aggregated at various temporal scales (30 min, 1 h, 2 h, 4 h, 6 h, 12 h and 24 h). Segmented models are particular cases of regression models where the *X*–*Y* relationship is piecewise linear, that is, regression with two or more segments connected at change points. We used two techniques, *segmented* [33] and *mcp* [34] (from the R packages of the same name), to detect change points in the ODBA–time relation. Both techniques are similar: iterating (generalized) linear models over possible locations of the change point(s) and returning a fit that minimizes the cost of the regression models. *Segmented* and *mcp* differ in how they solve the cost minimization function using a frequentist and Bayesian framework, respectively. While this implies that *mcp* is more computationally demanding, *mcp* presents the advantage of enabling hierarchical models with specific random effects that can allow the change point to vary within a group. We used a hierarchical model that included individuals in the experiment as a group. With both techniques, the number of change points for each segmented relationship must be specified *a priori*. We used one change point as we were interested in finding the most substantial change in activity, which we expected to occur somewhere along the disease course, between healthy and animal death. For both approaches, we extracted the change point value, slope, and certainty of the regression post-change-point.

Second, we derived the activity fingerprint along the ASF onset in the infected and contact animals (i.e. healthy, infection, contact, and viremia phases). We used the quartile classes of the ODBA frequency distribution, which provide a quantitative measure of the general activity of an animal over a defined period [30].

Third, to validate the observations obtained under laboratory conditions, we compared acceleration data from laboratory individuals with similar data obtained from semi-free and free ranging wild boar individuals. Semi-free and free ranging marked individuals were not infected with ASF, and based on the body conditions assessment performed during capture and recapture, these animals were considered healthy. Animals living in natural conditions allowed us to test the unambiguity of the signal observed in infected and contact animals. We hypothesized that semi-free and free ranging individuals would maintain a relatively constant level of activity, in contrast to the infected and contact groups, whose activity is expected to decrease after infection. We used the slope of the ODBA time series to test for this hypothesis, predicting that the slopes observed in the infected and contact groups are negative and steeper compared to animals in semi-free and free-ranging conditions. We tested this hypothesis for different observation time windows (30 min, 1 h, 2 h, 4 h, 6 h, 12 h and 24 h). We used the Bayes factor to assess evidence for our hypothesis [35]. Based on the Bayes theorem, this factor represents the ratio of the posterior probability of the tested hypothesis (i.e. slope in the infection phase being lower and negative compared to the healthy phase) to the posterior probability of the alternative hypothesis (i.e. slope in the infection phase being higher and positive compared to the healthy phase). We used the Savage–Dickey density ratio available in the *mcp* package [34] to approximate the Bayes factor [36].

Finally, for all observed animals (i.e. laboratory, semi-free and free ranging), we computed health curves, which we defined as the cumulative daily ODBA values. We observed the health curves of the animals under laboratory conditions 15 days before their death. For animals in semi-free and free-ranging conditions, we randomly selected 15 days during the entire tracking period. We considered a 15-day time window because it fitted the known course of ASF in wild boars, from infection to death [37,38]. We tested the cumulative mean and median ODBA values as health curve indicators. We predicted that the health curves of animals under semi-free and free-ranging conditions would demonstrate constant linear growth, and the health curves of infected animals would depart from linearity starting in the viremia phase.

## 3. Results

All laboratory animals tested positive and died following ASFV infection. The time of death for the infected animals (*n* = 3) was 7 days post-challenge, and for the contact animals (*n* = 9), the time of death ranged between 11 and 17 days after contact with the infected animals.

### (a) Segmentation analysis

**Figure 2.**
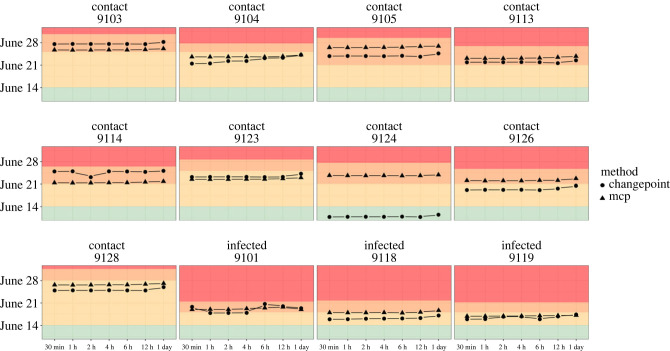
Detected change points for the infected and contact individuals in relation to the healthy (green area), infection or contact (yellow area) and viremia (orange area) phase for different lengths of aggregation of the acceleration data from windows of 30 min to an entire day. The red area indicates the period when animals died.

In 96% (23 out of 24, 1 *mcp* and 1 *segmented* analysis × 12 laboratory individuals) of the cases, the segmentation approach successfully detected and allocated a change point to a time post-infection or post-contact with infected animals (figure 2). In the case of contact animal ID 9124, the *segmented* approach wrongly attributed the change point to the healthy phase. For the change points that were adequately detected post-infection, there was a good match (in terms of detection timing) between the *mcp* and *segmented* approaches, independent of the aggregation window (figure 2). For five individuals, the change point was detected during the infection/contact phase, and six others during the viremia stage. For one individual, ID 9126, the two approaches detected a behavioural change at different phases (viremia phase for *mcp* and contact phase for the *segmented* approach) (figure 2).

### (b) Overall dynamic body acceleration and the infection course

**Figure 3.**
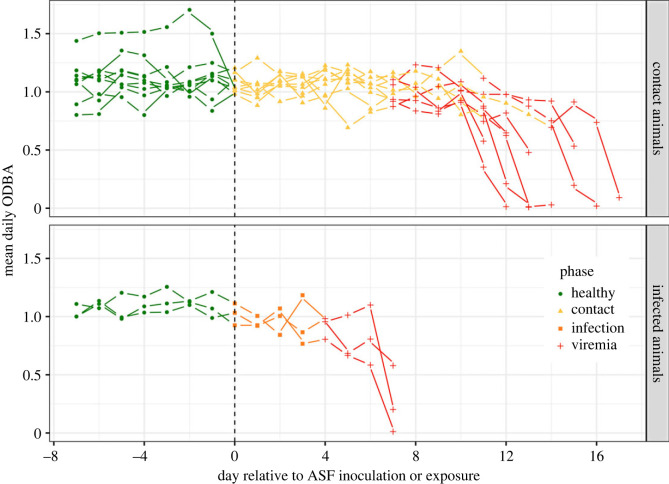
Variation in the mean daily ODBA per animal groups (infected and contact) and phases.

Among infected individuals (*n* = 3), the mean ODBA during the healthy phase, calculated over 8 days pre-infection, was 1.081*g* (s.d. = 1.14, range = 0.002–7.371) and 0.964*g* (s.d. = 1.084, range = 0–6.661) in the infection phase, and decreased to 0.754*g* (s.d. = 1.071, range = 0–6.156) after viremia was first detected. Among contact individuals, the mean ODBA during the healthy phase was 1.116*g* (s.d. = 1.171, range = 0–7.38) and 1.056*g* (s.d. = 1.125, range = 0.002–8.74) in the contact phase and decreased to 0.778*g* (s.d. = 1.06, range = 0–7.642) after viremia was first detected (figure 3).

At the daily level, *mcp* and *segmented* approaches indicate a relatively similar activity reduction after the change points, with 11.4% daily reduction for *mcp* and 16.4% for the *segmented* approaches (table 1).

**Table 1.** Summary statistic (as mean, lower and upper confidence intervals) of regression slopes after the changepoint detection for the two tested methods and the different aggregation windows. Note: for the *mcp* method which is based on Bayesian statistics, the slope mean is the posterior mean, the lower value is quantile of the highest-density interval (HDI) given in width, and the upper value is the upper quantile. *Rhat* is the Gelman–Rubin convergence diagnostic (acceptable if less than 1.1) and *n.eff* is the effective sample size, i.e. the number of independent samples produced by the MCMC algorithm.

method	period	mean	lower	upper	*Rhat*	*n.eff*
mcp	30 min	−0.0023	−0.0028	−0.0020	1.0132	327
	1 h	−0.0046	−0.0055	−0.0037	1.0051	354
	2 h	−0.0092	−0.0115	−0.0073	1.0089	387
	4 h	−0.0185	−0.0231	−0.0144	1.0042	359
	6 h	−0.0274	−0.0346	−0.0203	1.0016	400
	12 h	−0.0560	−0.0707	−0.0417	1.0086	399
	1 day	−0.1146	−0.1443	−0.0837	1.0029	368
segmented	30 min	−0.0032	−0.0054	−0.0010		
	1 h	−0.0057	−0.0101	−0.0014		
	2 h	−0.0088	−0.0124	−0.0051		
	4 h	−0.0234	−0.0398	−0.0070		
	6 h	−0.0529	−0.0979	−0.0078		
	12 h	−0.0787	−0.1246	−0.0328		
	1 day	−0.1192	−0.1687	−0.0697		

### (c) Fingerprint of the disease onset

**Figure 4.**
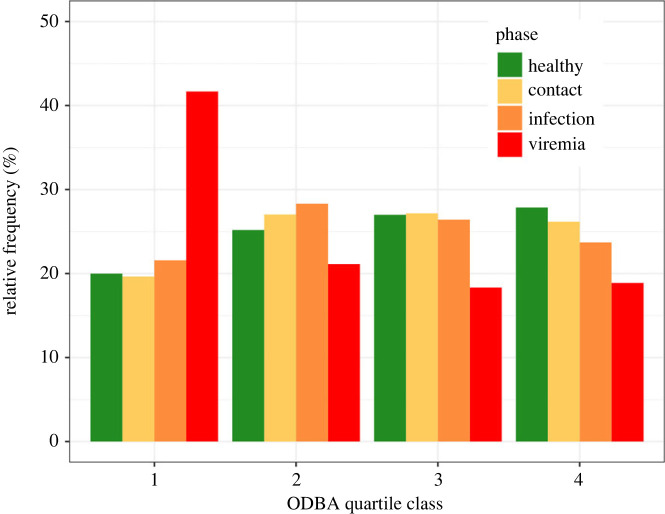
ODBA relative frequency distribution according to quartile classes for different observation phases (i.e. sum to 100% by phase).

When uninfected, we observed that laboratory wild boar had relatively well-distributed activity patterns (as described by the four quartile classes), with an increasing frequency from low activity (21% of ODBA bursts belonging to class Q1) to high activity (28% of ODBA bursts belonging to class Q4) (figure 4). When entering the infection and contact phases, wild boar activity patterns shifted to lower ODBA activity classes. Once viremia was detected, the wild boar mostly showed lower activity class behaviour (40% of the ODBA bursts belonging to class Q1) and much less high-class ODBA (18% of the ODBA bursts belonging to class Q4).

### (d) Comparison with semi-free and free-ranging animals

**Figure 5.**
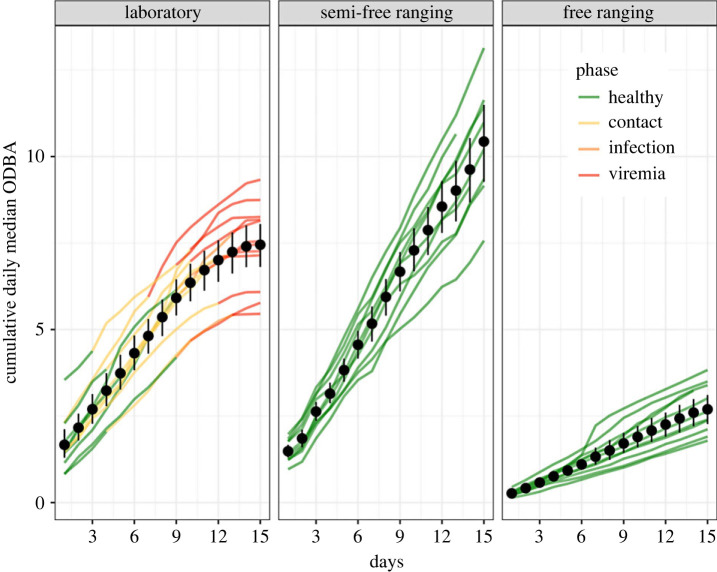
Comparison of cumulative daily ODBA between laboratory animals, semi-free and free ranging animals. Each line corresponds to one individual.

Infected and contact animals showed a prominent response due to disease onset and provided a clear signal of the presence of ASFV three to four days before death (figure 5). We found that the cumulative daily ODBA provided a good indication of the animal's health status over time (figure 5). When the animals were healthy, the cumulative daily ODBA showed a linear trend, and after infection, the linear trend tended toward an inflection point around the onset of viremia. The curve with the median values showed a clearer signal than that with the mean values (electronic supplementary material, figure S5), which was influenced by the occurrence of high ODBA values even in the latest disease phase.

Slopes from semi-free, free-ranging individuals were dissimilar to the slopes observed in infected and contact wild boars (*p* = 1, where *p* is the proportion of MCMC samples where slope_semi-free_ and slope_free ranging_ conditions were higher than slope_infected+contact_). This result was consistent for both techniques (*mcp* and *segmented*) and across all the tested aggregation windows (electronic supplementary material, table S1).

## 4. Discussion

We found that accelerometer sensors can detect sickness behaviours, specifically changes in wild boar activity patterns, associated with ASF infection. ASF infection caused a reduction in the activity of individuals during the viremia phase when the virus was first circulating in the blood. Through a comparison of infected individuals under laboratory conditions and those living under natural conditions, we demonstrated an unambiguous response of wild boars to ASF infection, as indicated by decreased activity levels. Our findings confirm the reliability of accelerometer technology in detecting such changes in activity levels, highlighting its potential for monitoring and controlling the spread of ASF in wild boar populations.

For all individuals in the infected and contact groups, we detected a clear decrease in activity associated with the onset of ASF. After the drop in activity, which for most individuals corresponded to the viremia phase, activity was reduced by approximately 10–20% daily. This reduction in activity and energy expenditure is in line with ananimal's metabolic rate response to pathogens and is a common behavioural response of animals as part of their sickness behaviour [19,39]. Our findings clarify previous observations on the impact of ASF on the activity of wild and domestic pigs. Using the same virulent strain, Arm07, a previous study did not find statistically significant differences in activity between infected and healthy individuals, although a decreasing trend was observed [40]. This difference from the present study can be explained by the use of different methods. Fernández-Carrión *et al*. [39] used computer vision with algorithm-based motion detection across frames. This approach primarily measures the animal displacement in a confined laboratory setting. By contrast, the accelerometer and ODBA metrics used in this study considered whole-body movement (specifically, head and ear movements). Therefore, ODBA is more likely to detect an overall change in activity even when animals are restricted to a small enclosure where large variations in displacement are unlikely to be observed.

Using video monitoring and accelerometers, Martínez-Aviles *et al*. [41] found a slightly lower reduction in activity (approximately 10%) in domestic pigs, probably because they used an attenuated strain of ASFV (Ken05/Tk1). Accelerometers are frequently used in livestock science and production to assess the health status and welfare of animals [16]. However, in wildlife disease ecology, attempts to remotely detect disease onset have been limited because of the challenge of working with free-living animals. Nevertheless, it has been shown that accelerometers have wide potential to detect sickness behaviour across different animal classes, including insects [18], reptiles [42], and large mammals [17]. Similarly, our results support the notion that accelerometer sensors can detect health issues in captive and free ranging animals.

Interestingly, we found a single case (animal ID 9124; electronic supplementary material, figures S1–S2) in which the segmentation technique did not associate a change point with disease onset. Animal ID 9124 was a contact animal, and as described in the Methods section, contact animals were placed in boxes with challenged animals. The observed reduction in the activity of animal ID 9124 prior to disease onset might be explained by the adaptation required by the individual to new housing and social environment. Hierarchical fights between individuals could also have triggered changes in behaviour, such as isolation from the group, which can result in lower activity levels over several days [43]. Compared with the *segmented* technique, the Bayesian *mcp* technique proved to be more robust in detecting the change in activity due to the disease, and less robust to the changes associated with the modification of the social environment. Thus, by accounting for random effects (i.e. individual identity), the *mcp* method commends itself with its superior performance in modelling and detecting varying change points [34]. Animal 9124 is a good reminder that changes in behaviour and states are context-dependent [18]; that is, an animal's activity level can simply be a response to other diseases or other noninfectious processes, such as hunger, trauma, injuries, manipulation, and human disturbances, as well as the wide potential of the *mcp* method to correctly detect signals in biotelemetry data [44]. Thus, we advocate the use of this technique, particularly in cases where groups of individuals are investigated.

Deploying sensors on an animal's ear triggers accelerometer data processing because the ear can move and indicate acceleration even when the rest of the body is immobile. This is often observed in suids, where individuals may flap their ears or have them nibbled on during social interactions [16]. However, the use of the ODBA as a composite metric that considers all three axes avoids the issue of sensor placement. Moreover, small changes in the sensor orientation do not pose a problem, as the observed acceleration decreases are mutually compensated between the axes [30]. Despite being relatively decoupled from the animal's locomotor body, accelerometers attached to the ear are highly correlated with the observed activity of the animal's body [45], and have demonstrated high accuracy in detecting changes in locomotion ability, such as lameness [46]. Future research should also investigate the impact of sensor attachment sites (including the ear and traditional GPS collars) on animal behaviour, as this topic has not been investigated to date.

**Figure 6.**
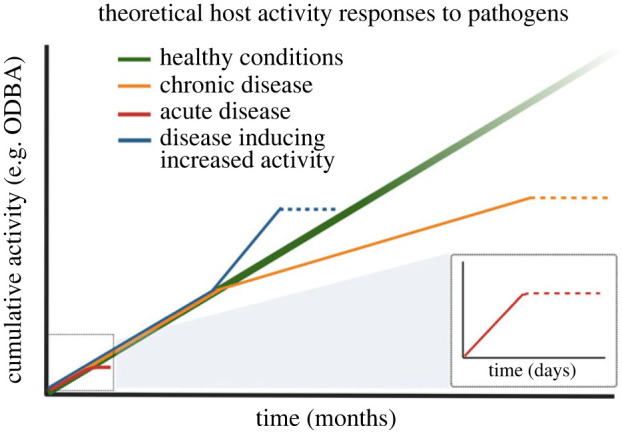
Proposed theoretical health activity curves according to various disease forms.

For broader applications of accelerometer sensors under natural conditions in free-ranging populations, further studies on behavioural changes induced by infection across host disease systems are required. Characterization of the different activity fingerprints that emerge is key to tracing how diseases develop in an organism, such as peracute, acute, subacute or chronic, and to understanding the different symptomatology involved (figure 6). Pathogens alter host movement not only by inducing energy loss but also by manipulating the host for their purposes [39,47,48]. For example, they can increase host movement to improve contact and spread to conspecifics [49,50] (figure 6).

In general, establishing fingerprints of animal health status across various taxa has considerable potential to enhance the inference ability based on observed tracking data. Animals face many challenges that compromise their health, and ultimately, their survival. Understanding the typical movement and activity pattern of an animal should move or be active under a certain status (e.g. when healthy or sick) can enable the retrospective analysis of large tracking databases to determine animal health status at different life stages. More empirical and quantitative evidence of activity changes after infection is needed across other host–disease systems to elucidate the variety of possible activity responses of an animal to disease. Therefore, assessing the specificity of animal responses in different host–disease systems is a key step toward the broader application of animal-attached sensors for sentinel surveillance [7]. Onboard processing of raw data is the way forward, so that only the relevant discrete processed information (e.g. daily mean and median ODBA) is extracted, while memory space and battery power can be optimized [51,52].

The time of first case detection is key to successfully controlling and eradicating emerging diseases. During the course of ASF, wild boars are not infectious before the viremia phase [53], suggesting that detecting and removing infected animals shortly after viremia detection could significantly affect disease dynamics. This shows the potential, and thus far largely overlooked, importance of accelerometer sensors in the management of emerging diseases. Novel surveillance approaches using resources such as mobile phones [54] and citizen science [55] have recently emerged to reduce the detection time of the first cases. At a time when biologging technologies have entered a golden age [11,56], providing us with unprecedented details regarding the life and behaviour of wild animals [13], it seems appropriate to further develop sentinel programmes in which animals are fitted with tracking devices. Recently, high-resolution biologgers deployed on sentinel animals have shown promising results in detecting poaching events [57] or earthquake risk in seismic zones [10]. Disease ecology could also benefit greatly from implementing more tracking technologies to improve animal detectability and to recover information on health status.

Following our experiment, it remains to be investigated how many animals would need to be equipped with sensors to provide a quantitatively reliable sentinel system suitable for early warnings. The rapid spread of ASF disease within social groups presents challenges in detecting individual infections. However, we believe that utilizing the methodology at various scales can serve as effective sensors, ranging from a coarse landscape level down to individual groups. Although deploying the methodology on a very large scale, such as nationwide, may be unlikely, we propose that accelerometer sensors can be strategically deployed in more sensitive and localized areas. For instance, they can be placed along the border of known infected zones or in buffer zones surrounding areas with a high pig farm density. The use of acceleration sensor eartags should certainly be of interest in preventing spillovers in domestic pigs and semi-wild farmed wild boars, which can easily be equipped with sensors.

The next generation of eartags can achieve increased energy efficiency, where eartag sensors would process data on board and send position alerts only in cases of suspected changes in acceleration profiles [51,58]. Thus, highly energetically efficient acceleration sensors can last for long periods. In the future, tags with onboard processing can also include a self-training algorithm that accounts for the idiosyncrasies of individual and local differences and reduces false positive alerts [52]. Finally, rolling out an animal-borne early warning system is facilitated by the ease of applying an eartag, its very low cost compared to collars, and to a larger extent, the economic losses incurred by an outbreak. Therefore, although it is currently difficult to estimate the proportion of the wild boar population that needs to be tagged, which certainly depends on the level of accuracy and coverage aimed for, it is clear that this technology is feasible.

## 5. Conclusion

We provide evidence that ASFV alters the activity of wild boars in a manner that is quantifiable using accelerometer sensors. These behavioural changes have profound implications because they can modify social and ecological interactions, ultimately influencing the disease dynamics. Therefore, understanding the impact of diseases on host behaviour will allow better forecasting of disease spread and the implementation of ad hoc surveillance approaches and thus rapid management actions in ASF disease. We argue that tracking technologies are underused tools that can greatly improve disease surveillance across multiple host–pathogen systems. To accomplish this, we call for closer collaboration among the disciplines of movement ecology, disease ecology, and veterinary science [47]. Our findings open the door to remote surveillance systems using biologging tools for the early detection and warning system of infectious diseases across various wild host–disease systems, particularly where the disease heavily impacts an animal's motion.

## Data Availability

The data and R code for analysis are accessible at Dryad [59]. The electronic supplementary material is accessible at Figshare [60].
